# Supplementary material for: A set of multi-entry identification keys to African frugivorous flies (Diptera, Tephritidae)
Source: Zookeys. 2014 Jul 24;(428):97–108. doi: 10.3897/zookeys.428.7366 (PMC4143993; doi:10.3897/zookeys.428.7366)
Supplement: Supplementary material 4 — Key to Capparimyia [file zookeys-428-097-s004.zip › SF4_ZooKeys_key to Capparimyia/lucid_player/help/help/best.htm]

Finding the Best feature - The Best function


# Finding the Best feature - The Best functions

Once one or more states have been chosen and the list of *Entities
Remaining* has been reduced, some features may be more useful for
the next step than others. For example, some features may be scored the
same for all entities remaining, in which case choosing a state of that
feature would not contribute to the identification.

Lucid3 has a *Best* algorithm
for determining which is the best feature to address at any stage during
an identification session. In general, a feature that would, on average,
cause the list of *Entities Remaining*
to be halved is a good feature to address.

There are two *Best* functions,
*Find Best* and *Sort
Best* (you can choose which *Best*
function to use from the *Best Mode*
item of the *Features* menu):

***Find
Best*** will determine which is the best feature to address next,
and jump to and open that feature in *Features
Available*. *Next Best* and *Previous Best*
 buttons on the toolbar allow navigation through the features
list, if the first feature nominated cannot be addressed.

***Sort Best***
will reorder the *Features Available*
list so that features are sorted from best to worst. After a *Sort
Best*, scan the top of the list for features that you can answer
most easily.

**Note** that Sort Best
only works using List View, as a tree of features cannot be sorted. See
the topic Trees
and Lists for more information.

Find the best feature to address next by clicking the *Best*
button  on the toolbar, or choosing *Find
Best* or *Sort Best* from
the *Features* menu.

If the list of entities in *Entities
Remaining* changes after choosing a feature as suggested by *Best*, it may be worth clicking the
*Best* button again to recalculate
the next best feature to address.

*Best* can be automated, so that
it is recalculated after each feature is addressed. Choose the option
*Automate*>*AutoBest*
from the *Features* menu. If you
need to choose several states from one feature when *AutoBest*
is on, hold down the *Control*
key when choosing the states � *AutoBest*
will be paused until the *Control*
key is released.
